# *Filifactor alocis* FtxA blocks inflammation and apoptosis pathways in monocytic cells

**DOI:** 10.3389/fcimb.2026.1745721

**Published:** 2026-03-23

**Authors:** Zeinab Razooqi, Kai Bao, Abdelbasset Yabrag, Naeem Ullah, Raviprakash T. Sitaram, Mark Lindholm, Mattias Pettersson, Anders Johansson, Georgios N. Belibasakis, Aftab Nadeem, Jan Oscarsson

**Affiliations:** 1 Department of Odontology, Umeå University, Umeå, Sweden; 2 Division of Oral Health and Periodontology, Department of Dental Medicine, Karolinska Institutet, Stockholm, Sweden; 3 Department of Molecular Biology and Umeå Centre for Microbial Research (UCMR), Umeå University, Umeå, Sweden

**Keywords:** apoptosis, extracellular vesicles, *Filifactor alocis*, FtxA, inflammation, periodontitis, RTX toxin, THP-1 cells

## Abstract

*Filifactor alocis* is an emerging oral pathogen, and approximately 50% of known *F. alocis* strains encode and express a Repeats-in-Toxin (RTX) protein, FtxA. FtxA appears to be associated with both progress and severity of periodontal disease. Mechanisms are not yet known but could be linked to increased loads of *F. alocis* in *ftxA*-positive strains. Here, we investigated mechanistic correlations based on FtxA-activity, as present in *F. alocis* cells and extracellular vesicles and as a recombinant protein, exploiting THP-1 macrophage-like cells. For this, we used the *ftxA*-expressing strain, ATCC 35896 (*ftxA*^+^), and *F. alocis* 148B-17U (*ftxA*^−^), which naturally lacks the *ftxA* gene. Using RNA sequencing analysis (RNA-Seq) and cytokine array analysis, we have pinpointed a role of FtxA in shifting host response toward immunosuppression, also inhibiting apoptosis and immune cell recruitment, and with a potential role in downregulating mitochondrial and oxidative phosphorylation pathways. Such role(s) could provide a plausible explanation why FtxA is associated with progress and severity of periodontal disease, and further studies on FtxA-host cell interactions might reveal novel potential therapeutic targets.

## 1 Introduction

Periodontitis destructs tooth support via interactions between microbial communities and the host immunity (Spahr and Divnic-Resnik, 2022). Historically associated with Gram-negative bacteria such as *Porphyromonas gingivalis* and *Aggregatibacter actinomycetemcomitans*, the Gram-positive anaerobe *Filifactor alocis* has been recently recognized as a key organism in modulating the immune environment in periodontitis (Aruni et al., 2014), peri-implantitis (Lee et al., 2017), and endodontic infections (Zehnder et al., 2017). The prevalence of *F. alocis* increases in severe periodontitis cases (Faisal and Ali, 2025).

Synergism appears to occur between *F. alocis* and *A. actinomycetemcomitans* in periodontitis, as judged by co-carriage, and on *A. actinomycetemcomitans* enhancing *F. alocis* accumulation, although depending on *F. alocis* strain it interacted with (Fine et al., 2013; Wang et al., 2013; Razooqi et al., 2024b; Razooqi et al., 2024a). *A. actinomycetemcomitans* may thereafter be outcompeted by *F. alocis* in deeper periodontal pockets (Dahlén et al., 2014; Razooqi et al., 2022), indeed mainly at sites with deep periodontal pockets/greater attachment loss (Faisal and Ali, 2025). Recently, *F. alocis* was shown to promote a shift from a homeostatic oral microbial community toward a dysbiotic state (Vashishta et al., 2025). The cellular mechanisms behind this shift remains unknown but is known to require the presence of Toll-like receptor 2 (TLR2).

*F. alocis* virulence includes manipulating neutrophils and extending their lifespan (Miralda et al., 2022; Ozuna et al., 2022). This could promote bacterial survival by preventing neutrophil extracellular trap (NET) formation (Miralda et al., 2022). This can inhibit phagosome maturation, thereby reducing reactive oxygen species (ROS) production and impairing neutrophil-mediated bacterial killing. *F. alocis* may hence proliferate in the oxidative-stress-rich environment of the periodontal pocket. Potential virulence factors include a superoxide reductase (FA796) and a hypothetical protein FA51, involved in the ROS detoxification pathway, suggesting a role in ROS resistance (Aja et al., 2021). Moreover, *F. alocis* can affect macrophages by delaying apoptosis, and thereby prolonging the inflammatory cascade, hence promoting periodontitis (Nogueira et al., 2021). Additionally, one *F. alocis* complement system inhibitory protein, FACIN, appears to be involved in arginine metabolism (Jusko et al., 2016).

*Filifactor* toxin A (FtxA) is a Repeats-in-Toxins (RTX) protein encoded and produced in 50% known *F. alocis* (Oscarsson et al., 2020; Bao et al., 2022b). Interestingly, *ftxA* is associated with both progress and severity of periodontitis (Razooqi et al., 2024b; Razooqi et al., 2024a). The mechanism(s) is not known, albeit potentially linked to enhanced loads of *F. alocis* (Razooqi et al., 2024b). RTX proteins are predominantly found in Gram-negative bacteria (Linhartova et al., 2010), making *FtxA* one of the first RTX proteins identified in a Gram-positive oral pathogen. Interestingly, the closest homologue to FtxA among potential oral pathogens is a Ca^2+^-binding, RTX-related protein of *Eubacterium yurii* strain ATCC 43714 (SKC68031), which shows ≈35% amino acid sequence identity (Oscarsson et al., 2020). This species is detected in infected root canals of traumatized teeth (Manoharan et al., 2020).

The FtxA-host cell interaction is unknown, albeit potential cytopathic effects could be considered analogously to the RTX-leukotoxin (LtxA) of *A. actinomycetemcomitans* (Belibasakis et al., 2019). FtxA has been identified in extracellular vesicles (EVs; also referred to as membrane vesicles [MVs] or bacterial membrane vesicles [BMVs]) from *F. alocis* (Kim et al., 2020; Bao et al., 2022a). It is hitherto not known whether FtxA or any EV-associated protein(s) might have induced the immunostimulatory effects of vesicles on human monocytic and oral keratinocyte cells (Kim et al., 2020), or contributed to the induction of osteoclastogenesis and systemic bone loss through TLR2 (Song et al., 2020; Kim et al., 2021). Here, we opted to investigate the mechanism(s) on how *F. alocis* via FtxA acts on human macrophage-like cells, which could potentially explain the association of *F. alocis* with the progression of periodontal disease.

## 2 Materials and methods

### 2.1 Bacterial strains and growth conditions

*F. alocis* ATCC 35896 (*ftxA*^+^; also referred to as CCUG 47790) was originally isolated from gingival sulci of patients with gingivitis or periodontitis (Cato et al., 1985; Jalava and Eerola, 1999) and was selected for this work on the basis of encoding and expressing FtxA (Bao et al., 2022b). *F. alocis* 148B-17U (*ftxA*^−^) was isolated from a case of periodontitis (Oscarsson et al., 2020) and chosen to represent an FtxA non-producing strain. The strains are otherwise generally comparable regarding growth characteristics and baseline protein profiles (Oscarsson et al., 2020; Bao et al., 2022b). The strains were cultivated anaerobically (10% H_2_, 5% CO_2_, 85% N_2_) on fastidious anaerobe agar (FAA; Neogen^®^, Heywood, UK) at 37 °C for approximately 7 days (Oscarsson et al., 2020). Whole-cell extracts of *F. alocis* strains were harvested from FAA plates in 1× phosphate-buffered saline (PBS).

### 2.2 Extracellular vesicle isolation from *F. alocis*

EVs were isolated from triplicate samples of *F. alocis* ATCC 35896 (*ftxA*^+^) and 148B-17U (*ftxA*^−^), harvested from 20–25 agar plates in PBS, obtaining an OD_600nm_ of 1-3, and subsequently ultracentrifuged (Lindholm et al., 2020; Metsäniitty et al., 2024). In brief, EV pellets were washed twice with PBS (85,000 × *g*; 2 h, 4 °C) using a 70-Ti rotor (Beckman Instruments Inc.), resuspended in ≈200 μl PBS, and then used for the EV preparation. The yield of vesicles was estimated by determining protein concentrations using a NanoDrop 1000 spectrophotometer (Thermo Fisher Scientific), and preparations were assessed for EV particle concentration using Nanoparticle Tracking Analysis software (NanoSight Ltd.). Sample EVs were spotted for absence of contamination on FAA in air supplemented with 5% CO_2_ at 37 °C for 3 days.

### 2.3 Electron microscopy

Analyses were carried out at the Umeå Core Facility for Electron Microscopy (UCEM). For transmission electron microscopy of OMV preparations, 3.5 µl of samples was applied to glow discharged formvar and carbon-coated Cu-grids. The grids were washed and negatively stained in 1.5% uranyl acetate for 2 × 15 s. Samples were examined with a Talos 120C transmission electron microscope (FEI, Eindhoven, The Netherlands) operating at 120 kV. Micrographs were acquired with a Ceta 16M CCD camera (FEI, Eindhoven, The Netherlands) using Velox version 2.14.2.40 från Thermo Fisher Scientific (Eindhoven, The Netherlands). For scanning electron microscopy, small pieces of agar containing bacterial colonies were fixed in 2.5% glutaraldehyde in 0.1 M sodium cacodylate buffer (pH 7.4) at 4 °C overnight, and then dehydrated in graded series of ethanol, critical point dried, and metal-coated (4 nm). The morphology of the samples was analyzed with a field-emission scanning electron microscope (Zeiss Merlin FESEM).

### 2.4 Proteomics identification of vesicle-associated FtxA, and pilin-associated proteins

EV protein concentrations were measured using Qubit Protein Assay (Thermo Fisher Scientific). Vesicle preparations (triplicates from each strain; ≈5 µg protein each) were digested for mass spectrometry using PreOmics^®^ iST Kit (PreOmics, Germany), briefly resuspended in 100 µL of lysis buffer, provided in the kit, incubated at 95°C for 10 min, and treated with high-intensity focused ultrasound (HIFU). For enzymatic digestion, 50 µL of PreOmics Digestion Mix was added, following incubation at 37°C with shaking (60 min). Digestion was halted by adding 100 µL stop solution and processed through the iST filter cartridge, where peptides were retained by the membrane and the remaining solution removed via centrifugation at 3,800 × *g*. Peptides were then washed, eluted, dried, and reconstituted in 20 µL injection buffer (3% acetonitrile, 0.1% formic acid). Frozen peptides were reconstituted in 3% acetonitrile with 0.1% formic acid and analyzed using an Orbitrap Fusion mass spectrometer (Thermo Fisher Scientific). Data-independent acquisition (DIA) spectra were processed with DIA-NN v1.8.2, using a library-free approach. FtxA (E8RK95) was extracted based on a custom concatenated database of UniProt *F. alocis* proteomes, along with in-house generated *F. alocis* proteomes derived from the genomic sequences of *F. alocis* strains ATCC 35896 and 148B-17U (Oscarsson et al., 2020), and pilin- and fimbriae-associated proteins were extracted in addition. Carbamidomethylation of cysteine was set as a fixed modification, whereas acetylation (protein N-terminus), oxidation (methionine), and methionine loss (protein N-terminus) were specified as variable modifications.

### 2.5 Recombinant FtxA protein production

FtxA protein was produced in *E. coli* following the procedures described earlier (Oscarsson et al., 2020). Liquid chromatography-tandem mass spectrometry (LC-MS/MS) was used to confirm the identity of the obtained FtxA, via the ACQUITY I-Class XEVO G2 XS high-resolution UPLC/MS system from Waters (Milford, MA, USA). Five microliters of protein sample was injected on a BioResolve™ RP mAb Polyphenyl, 450-Å, 2.7-µm column (Waters, Wexford, Ireland), using a short gradient of Milli-Q ultrapure water and acetonitrile, with 0.1% difluoroacetic acid. Mass spectrometry of eluted proteins was done acquiring from 500 to 3,000 m/z, and the multiple charged clusters were deconvoluted with MaxEnt1 software (part of the MaxEnt suite in MassLynx version 4.2; Waters™) to obtain neutral mass of the intact protein.

### 2.6 Cultivation of human monocytic leukemia THP-1 cells

Human myeloid monocytic THP-1 (ATCC^®^ TIB-202™) cells were maintained in Dulbecco’s modified Eagle medium (DMEM) (Sigma-Aldrich) essentially as earlier described (Yabrag et al., 2025). Prior to experimental treatment, cells were supplemented with 10% fetal bovine serum (FBS) and 1% penicillin/streptomycin at 37 °C with 5% CO_2_. Prior to exposure to test agents, the THP-1 cells were treated with phorbol 12-myristate 13-acetate (PMA) (at 100 ng/ml) for 48 h to differentiate them to a macrophage-like phenotype and thereafter washed and harvested in PBS. This macrophage-like phenotype of THP-1 cells was used in all cell experiments described in the present work and referred to as THP-1 cells.

### 2.7 THP-1 cell toxicity assays

THP-1 cells (1 × 10^4^ in DMEM) were infected with *F. alocis* cells, which were prior harvested in PBS, at multiplicities of infection (MOI) of 2, 20, and 200, respectively. The THP-1 cells were also incubated with EVs (at 3.5 μg/μl) and purified FtxA (at 0.25, 0.5, and 1 μg/ml). Triton X-100 (0.1%) was used as a positive control for lysis, and as a negative control, untreated THP-1 cells were utilized. All incubations were carried out in DMEM supplemented with 10% fetal bovine serum (FBS) at 37 °C with 5% CO_2_ for 24 h. Following the experimental treatment, propidium iodide (PI, at 3 µg/ml) was added to identify dead cells, following image acquisition every hour for 24 h with the Spark^®^ Cyto imaging system (Tecan). Images were analyzed by Cellpose 3 (Stringer and Pachitariu, 2025). A customized Python code was developed for batch processing of images. Parameters for image processing included the Cyto3 model with an 18-pixel diameter for bright field images and an 18-pixel diameter with a Gaussian smoothing filter of 6 pixels for propidium iodide-stained cells. The total number of PI-positive dead cells was normalized against the total number of cells.

### 2.8 THP-1 cell treatments for RNA-Seq and cytokine array analysis

THP-1 cells (1 × 10^5^ in DMEM) in 24-well plates at 37°C were treated with EVs (at 3.5 μg/ml) or purified FtxA (at 1 μg/ml) for 4 h. The 4-h timepoint was selected to capture early cytokine release while limiting later secondary effects (Bradburne et al., 2008; Unuvar Purcu et al., 2022). This concentration of EVs and FtxA, respectively, selected in these experiments was based on the absence of an apparent cytotoxic effect to the THP-1 cells, and still to be within the range of biologically relevant doses regarding EVs (Nguyen et al., 2020), and as judged by assessment of another non-lytic RTX-toxin, CyaA (Ross et al., 2004). After the experimental treatment, the cells were collected by transferring them to Eppendorf tubes and centrifuged at 15,000 rpm for 10 min. Cells and supernatants were taken to further analysis. Cells were taken for RNA-Seq (2.9), and supernatants for cytokine array (2.10) analysis, respectively.

### 2.9 RNA sequencing analysis

Total RNA was extracted using the RNeasy Mini Kit (Qiagen). Essentially as earlier described, library preparation and sequencing was carried out by BMKGene, with a paired-end protocol and read length of 150 nucleotides (PE150), generating 20 million reads per sample (Corkery et al., 2021; Nadeem et al., 2021). RNA-Seq analysis was performed with integrated Differential Expression and Pathway analysis (iDEP) (Ge et al., 2018). Briefly, differential gene expression (DEGs) analysis was performed from count matrix. Genes with adjusted P<0.05 and log2 fold changes more than 1 were considered differentially expressed, as indicated in the volcano plots. The DEGs among the *ftxA*^+^ and *ftxA*^−^ EVs, and purified FtxA, compared with the vehicle, *i.e.*, PBS-treated controls, were further described in a Venn diagram. A heatmap was generated from the top 1,000 DEGs in *ftx*A^+^ and *ftxA*^−^ EVs, and purified FtxA, compared with the vehicle-treated controls. In addition, the genes were functionally characterized using the gene set enrichment analysis (GSEA), which was performed with the GSEA software (Broad Institute, San Diego, USA; version 4.3.3). For enrichment score calculation, all genes were ranked based on the signal-to-noise metric (Subramanian et al., 2005). An enrichment score of 0.2 was used as the cutoff.

### 2.10 Cytokine array

THP-1 cell supernatants collected as above were profiled against 20 inflammatory cytokines, including interleukins, TNF-α, and interferons. For this, a RayBiotech Quantibody^®^ array was implemented (Ovine Cytokine Array Q20; catalog number QAO-CAA-20-1). This assay was selected on the basis on its documented cross-reactivity with human samples (Zhai et al., 2008; Toh et al., 2009; Vargas-Inchaustegui et al., 2010; Jonnalagadda et al., 2012). As controls, unstimulated cells, and unused growth medium, were used.

### 2.11 Statistical analysis

ANOVA of the cytokine array data was done in collaboration with Björn Tavelin, statistician at Department of Radiation Sciences, Umeå University. Unless specified otherwise, data are expressed as means ± standard deviation (SD) on the basis of at least three independent experiments. Means were compared using the two-tailed Students t-test. *P* values of less than 0.05 were regarded as statistically significant.

### 2.12 Image processing

Images for figures were assembled using Adobe Photoshop (24.4.1; Adobe, San Jose, CA, USA).

### 2.13 Ethical considerations

All procedures were conducted in accordance with the guidelines of the local ethics committee at the Medical Faculty of Umeå University, which are in accordance with the Declaration of Helsinki (75th WMA General Assembly, Helsinki, October 2024).

## 3 Results

### 3.1 Extracellular vesicle isolation from *F. alocis* strains ATCC 35896 and 148B-17U

**Figure 1:**
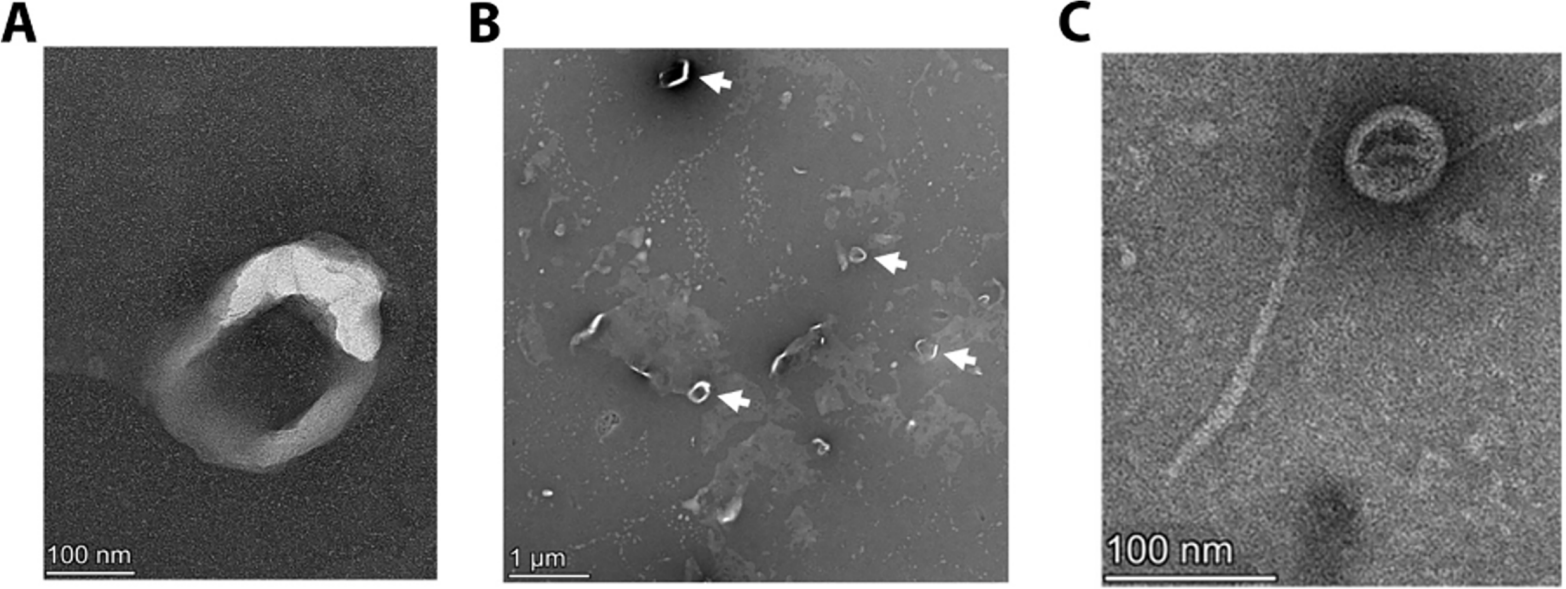
Electron micrographs of *F. alocis* strains and their released extracellular vesicles. Transmission electron micrographs of EVs released from ATCC 35896 (*ftxA*^+^) **(A, B)**, and 148B-17U (*ftxA*^−^) **(C)**, respectively. The arrows in **(B)** indicate examples of vesicles. Scale bars are shown in lower left corners.

EVs were isolated from *F. alocis* ATCC 35896 (*ftx*A^+^) and 148B-17U (*ftxA*^−^), obtaining an overall similar yield (mean protein concentration in the preparations of EVs from ATCC 35896 25.2 μg/ml, and 148B-17U 24.1 μg/ml). To characterize vesicle presence and morphology, transmission electron microscopy (TEM) was performed. TEM images showed the presence of spherical vesicles with a bilayer membrane, ranging in size approximately from 50 to 200 nm in diameter, obtained from both strains (**Figure 1**). The vesicles appeared as electron-dense structures, and we concluded that both strains assessed produced EVs under the growth conditions used. The filament(s) observed in panel 1C is most likely pilin-based according to the LC-MS/MS analysis of the vesicle preparations (**Supplementary Table 1**), consistent with previous studies (Blackburn et al., 2021; Kretschmer et al., 2023).

### 3.2 Identification of FtxA in extracellular vesicles of strain ATCC 35896

LC-MS/MS on *F. alocis* EVs was conducted to confirm presence of FtxA (E8RK95) from ATCC 35896 (*ftxA*^+^) and absence in 148B-17U (*ftxA*^−^). This revealed identification of FtxA in EVs of ATCC 35896 (several peptides were identified; **Supplementary Table 2**), whereas in contrast, FtxA was absent in vesicles from strain 148B-17U.

### 3.3 FtxA or as associated with extracellular vesicles does not kill THP-1 cells

**Figure 2:**
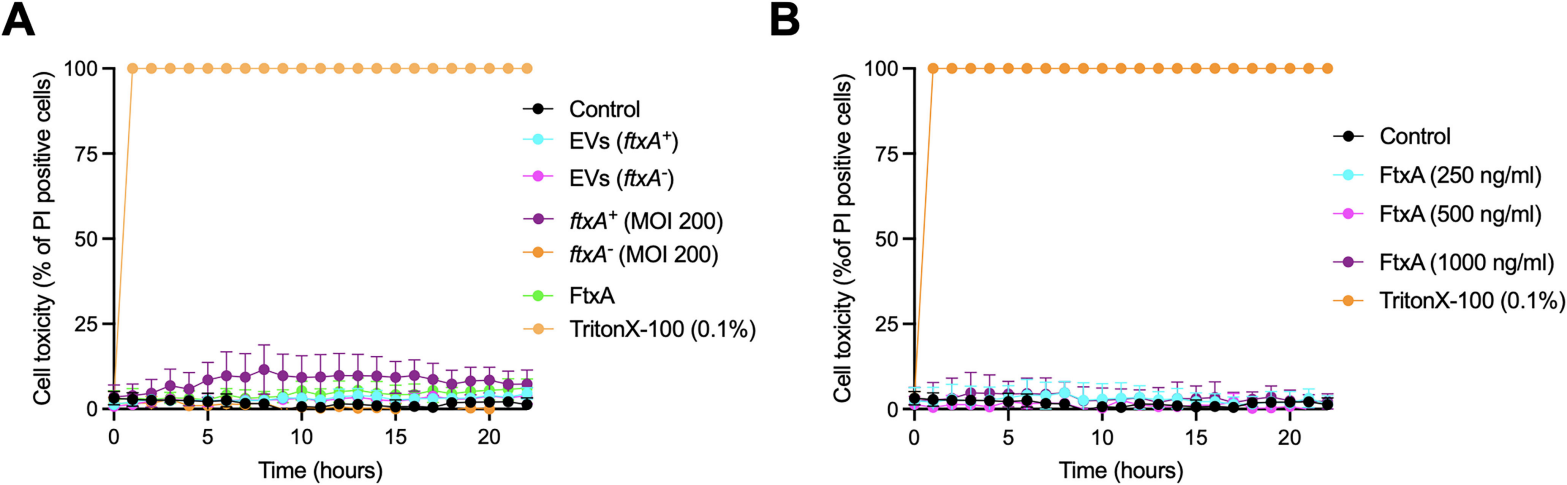
Kinetic analysis of potential FtxA-dependent THP-1 cell toxicity over time (hours). **(A)** THP-1 cell toxicity in response to EVs of ATCC 35896 (*ftxA*^+^) or 148B-17U (*ftxA*^-^), as stimulated with whole cell extracts (MOI 200) obtained from the same *F. alocis* strains, respectively, and with recombinant FtxA. **(B)** Monitored THP-1 toxicity upon increasing concentrations of recombinant FtxA. Cell toxicity is expressed as the percentage of propidium iodide (PI)-positive cells, minus background readouts (untreated THP-1 cells; Control). Triton X-100 (0.1%) was used as a positive control for cell lysis. Shown are the means from three independent experiments. For each time point, P<0.001 between individual readouts for experimental treatments.

To determine if presence of FtxA may provoke cell lysis, we assessed exposed THP-1 cells to EVs and whole-cell extracts, respectively obtained from *F. alocis* strains ATCC 35896 (*ftxA*^+^) and 148B-17U (*ftxA*^−^) (**Figure 2A**), or with increasing concentrations of recombinant FtxA (**Figure 2B**). This revealed no apparent cytotoxic response(s), consistent with the notion that FtxA was not killing the THP-1 macrophage-like cells. The figure indicates results using *F. alocis* whole-cell extracts at MOI 200. Also, *F. alocis* whole-cell extracts at MOI 2 and 20 were tested in this assay, indicating no apparent toxic effect (data not shown).

### 3.4 FtxA-associated immune response signaling suppression in THP-1 cells

**Figure 3:**
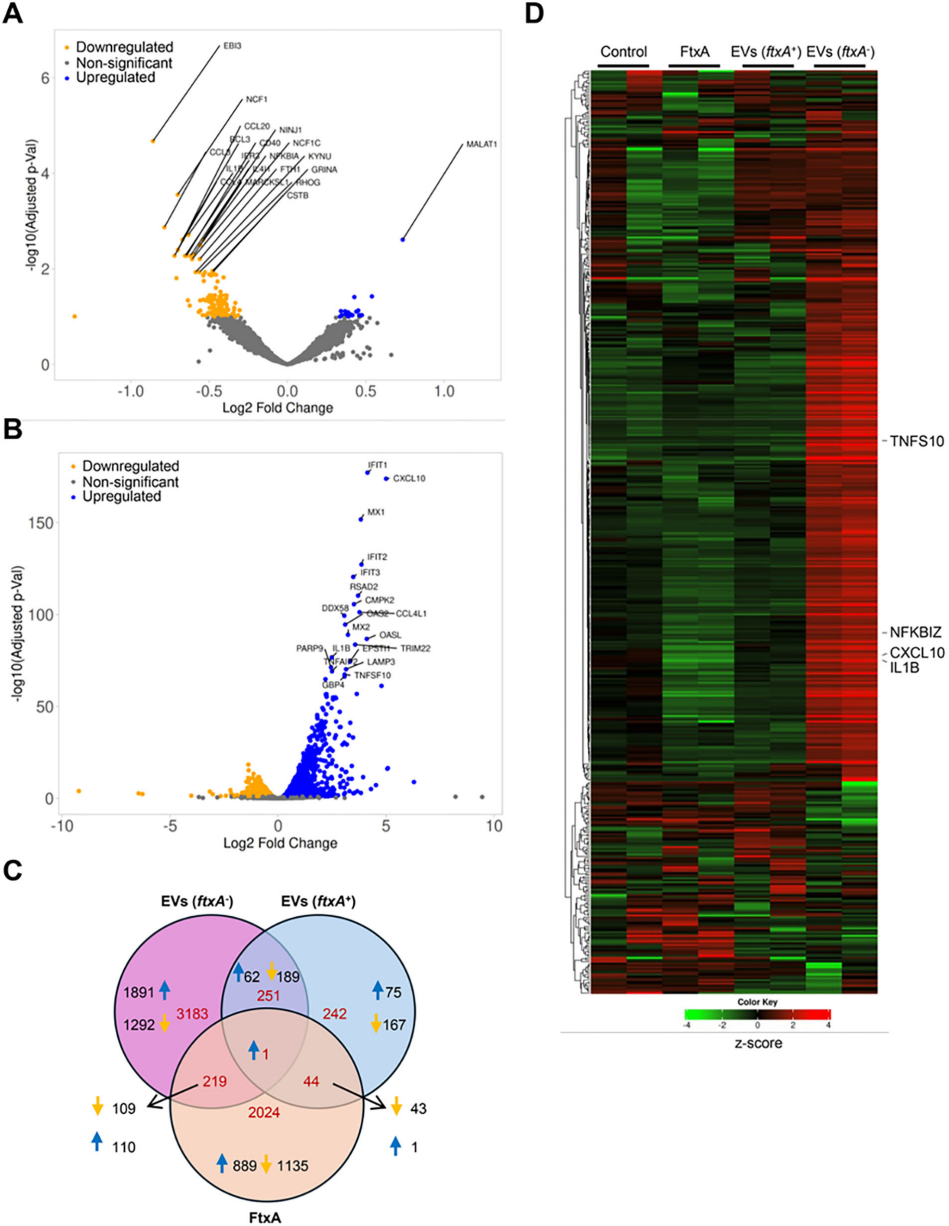
FtxA suppresses the immune response. THP-1 cells were exposed to *F. alocis ftxA*^+^ and *ftxA*^−^ EVs (3.5 μg/ml) and recombinant FtxA (1 μg/ml), respectively, for 4 h, followed by RNA-Seq analysis of two 4-h replicates. THP-1 cells treated with vehicle (PBS) were used as a control. Volcano plot indicating differential gene expression in THP1 cells treated with **(A)** *ftxA*^+^ EVs and **(B)** *ftxA*^−^ EVs. Top 20 differentially expressed genes are highlighted. **(C)** Venn diagram showing the distribution of differentially expressed genes in THP1 cells exposed to EVs (*ftxA^+^* and *ftxA^−^*), or recombinant FtxA. **(D)** Heatmap displaying differential gene expression in THP1 cells exposed to EVs (*ftxA^+^* and *ftxA^−^*), or recombinant FtxA. The genes involved in the immune response are indicated in the heatmap and are upregulated in the cells treated with *ftxA*^−^ EVs and downregulated upon exposure to either *ftxA*^−^ EVs or recombinant FtxA.

**Figure 4:**
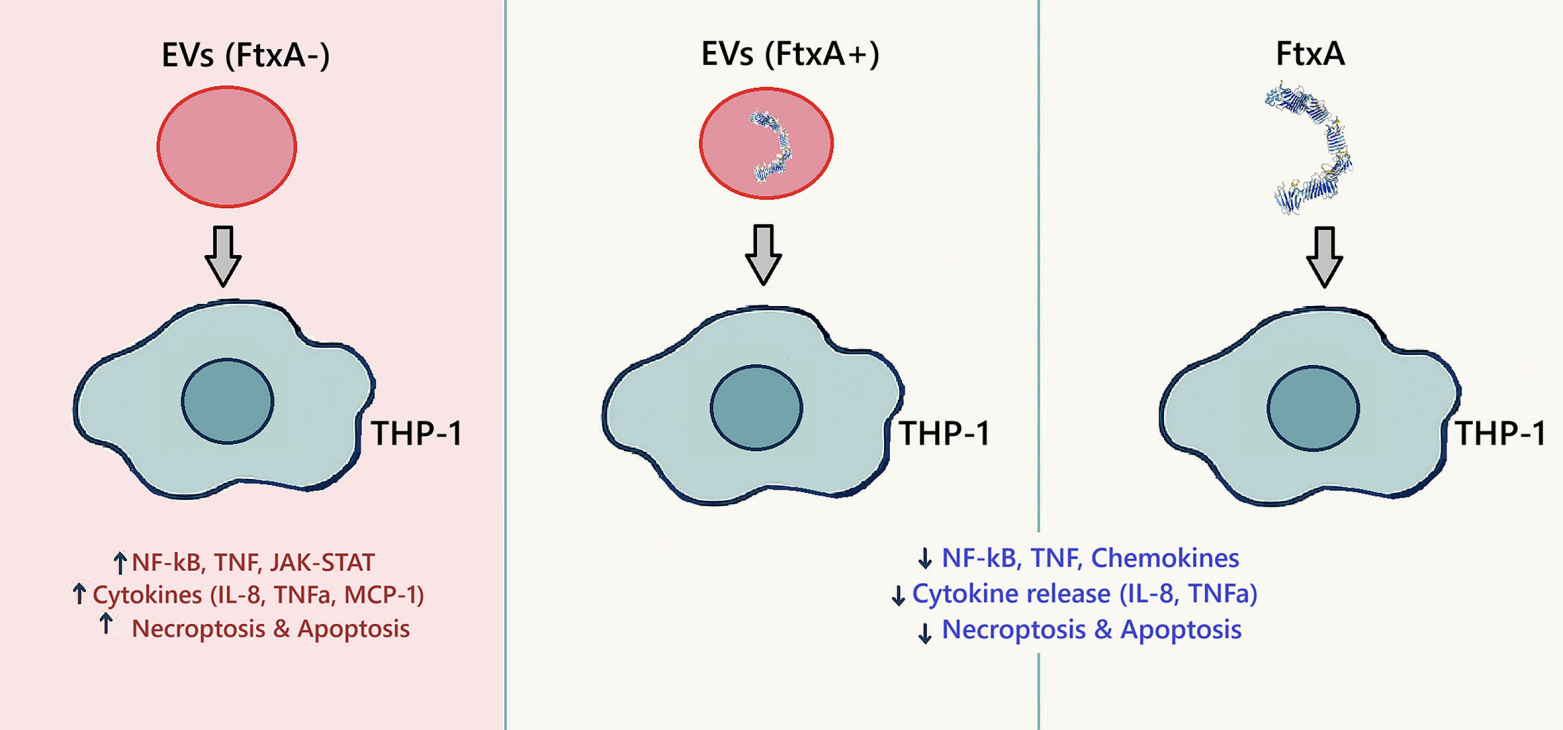
FtxA-dependent THP1 cell modulation responses to *F. alocis* EVs from ATCC 35896 (*ftxA*^+^), 148B-17U (*ftxA*^−^), and recombinant FtxA, after 4 h of stimulation. Key pathways up- and downregulated as determined by RNA-Seq are highlighted in red and blue text, respectively.

To assess the potential role of FtxA in virulence, we next monitored patterns of differentially expressed genes in exposed relative to untreated THP-1 cells using RNA-Seq, regarding biological processes (relevant pathways as delineated in **Supplementary Tables 3**, **4**, respectively). For this, cells were exposed to EVs from *F. alocis* ATCC 35896 (*ftxA*^+^), and 148B-17U (f*txA*^−^), and recombinant FtxA protein, respectively for 4 h. As displayed in **Figures 3**, **4**; **Supplementary Figure 1**, incubating THP-1 cells with *ftxA*^+^ EVs and FtxA protein revealed predominantly downregulated immune pathways, including cytokine–cytokine receptor interactions and NF-κB and TLR signaling (adjusted *P* < 0.001).

Expression of genes involved in IL-1β and IFN-γ responses were downregulated, with reductions in chemokine signaling pathway and leukocyte chemotaxis. Additionally, exposure by ATCC 35896 EV downregulated genes linked to ATP synthesis-coupled electron transport is suggestive of mitochondrial dysfunction. Exposure to purified FtxA potentiated a similar and more pronounced immunosuppressive transcriptome, with strong suppression of NF-κB, TNF-α, chemokine signaling, and cytokine–cytokine receptor interactions. Pathways associated with cytoskeletal organization were also reduced, consistent with impaired migration and altered cell morphology and metabolic disruption. Interestingly, there was increased expression of a long non-coding RNA (LncRNA) and Metastasis Associated Lung Adenocarcinoma Transcript 1 (*MALAT1*) in the THP-1 cells stimulated with EVs from the *ftxA*^+^ strain (**Figure 3A**). Using EVs from 148B-17U (*ftxA*^−^), in contrast, triggered a highly pro-inflammatory signature, characterized by strong upregulation of CXCL10, IL-1β, and TNFSF10 (**Figure 3B**). Comparative analysis of differentially expressed genes (DEGs) using a Venn diagram showed that multiple genes were differentially regulated in the THP-1 cells by EVs from the *ftxA^+^* and *ftxA*^−^ strains, or by purified FtxA (**Figure 3C**). Among these DEGs, EVs from the *ftxA^+^* and *ftxA*^−^ strains shared 251 genes, of which 62 were upregulated and 189 were downregulated. EVs from the *ftxA*^+^ strain and purified FtxA shared 44 genes, of which 43 were downregulated and only one was upregulated. Notably, among the top downregulated genes shared by *ftxA^+^* EVs and purified FtxA were key immune response genes, including IL-1β, TNF-α, and CXCL8 (**Figure 3B**; **Supplementary Figure 1**). Separately, purified FtxA specifically regulated 2,024 genes, with 889 upregulated and 1,135 downregulated (**Figures 3C, D**; **Supplementary Figure 1**). Meanwhile, EVs from the *ftxA^−^ F. alocis* strain differentially regulated 3,183 genes, the majority of which (1,891) were upregulated and 1,292 were downregulated (**Figure 3C**). Moreover, heatmap analysis (**Figure 3D**) indicated a strong upregulation of immune response genes in cells treated with *ftxA^−^* EVs. In line with this, key immune genes such as CXCL8 and IL-1β were upregulated by *ftxA^−^* EVs but were downregulated in THP1 cells treated with either *ftxA^+^* EVs or purified FtxA protein (**Figure 3D**; **Supplementary Figure 1**). The pathway analysis using gene set enrichment analysis (GSEA) suggested an upregulation of TNF-α, NF-κB, Janus kinase–signal transducer and activator of transcription (JAK-STAT), and cytokine–cytokine receptor interaction pathways, in cells treated with *ftxA*^−^ EVs. Cell death processes such as necroptosis were also activated, highlighting the immunostimulatory nature of *F. alocis* EVs lacking FtxA. In conclusion, and as summarized in **Figure 4**, FtxA *per se* and as associated with EVs appeared to act as a primary driver of an immunosuppressive transcriptome to attenuate the immune response.

### 3.5 FtxA-dependent suppression of apoptosis and cell death–related biological processes

**Figure 5:**
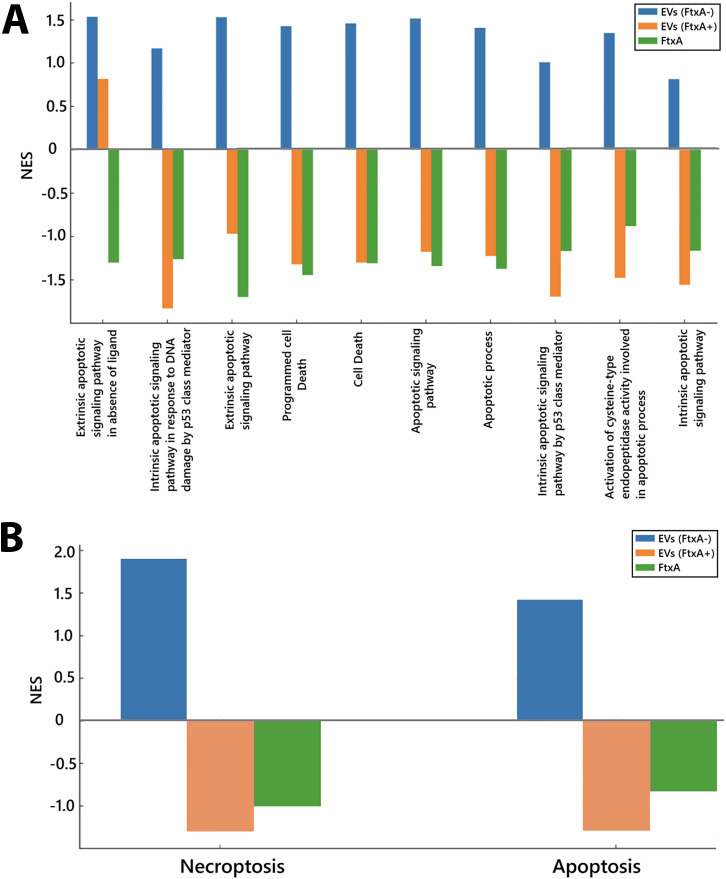
**(A)** Comparison of apoptosis-related biological processes in THP-1 cells stimulated with *F. alocis* EVs or purified FtxA. Gene set enrichment analysis of RNA-seq data shows that EVs from the *ftxA*^-^ strain 148B-17U (blue) upregulated multiple apoptosis-related pathways, including extrinsic and intrinsic apoptotic signaling and programmed cell death. In contrast, EVs from the *ftx*A^+^ strain (orange), as well as purified FtxA (green) predominantly downregulated these pathways, indicating a suppressive effect on host cell apoptosis. **(B)** KEGG pathway enrichment analysis of apoptosis and necroptosis assessing EVs from ATCC 35896 (*ftxA*^+^) and 148B-17U (*ftxA*^−^), and recombinant FtxA, respectively. Negative enrichment scores (NES) are indicated in both panels.

Gene set enrichment analysis highlighted a control of cell death pathways, which appeared to be linked to FtxA (**Figure 5A**; **Supplementary Table 3**). EVs from 148B-17U (*ftxA*−) upregulated apoptosis-associated processes (NES > +1.0), including extrinsic apoptotic signaling, apoptotic mitochondrial changes, p53-mediated apoptotic signaling, and programmed cell death. In contrast, EVs from ATCC 35896 (*ftxA*^+^) and especially FtxA *per se* suppressed apoptotic pathways, with negative enrichment scores (NES < –1.5), suggesting inhibition of host cell death responses. Hence, while EVs from the strain lacking *ftxA* induced pro-apoptotic transcriptional programs, EVs from ATCC 35896 and especially FtxA *per se* appeared to act to inhibit apoptosis, potentially prolonging host immune cell survival. This divergence was further corroborated by Kyoto Encyclopedia of Genes and Genomes (KEGG) analysis (**Figure 4B**; **Supplementary Table 4**), where 148B-17U (*ftxA*^-^) EVs strongly induced both necroptosis and apoptosis (NES > 1.4), whereas EVs from ATCC 35896 (*ftxA*^+^) and purified FtxA downregulated these pathways.

### 3.6 FtxA-dependent suppressive effect on cytokine expression

**Figure 6:**
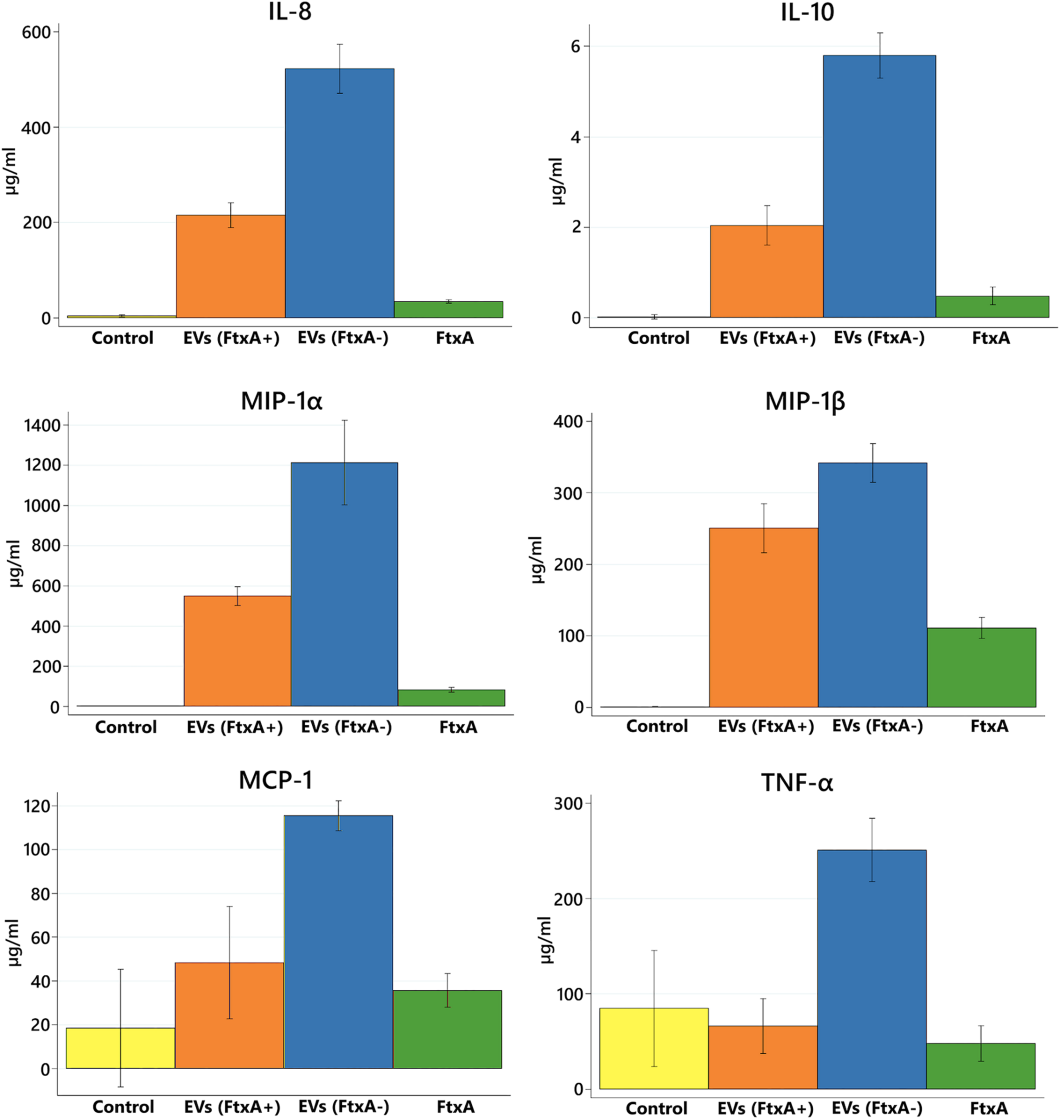
Cytokine array analysis of THP-1 cell supernatants (two biological replicates) after stimulation with EVs from the *F. alocis* strains ATCC 35896 (*ftxA*^+^), 148B-17U, (*ftxA*^-^), and FtxA recombinant protein, respectively. Expression of pro- and anti-inflammatory cytokines (TNF-α, IL-8, IL-10, MIP-1α, MIP-1β, and MCP-1) is shown. A significant reduction in the secretion of TNF-α, IL-8, IL-10, MIP-1α, MIP-1β, and MCP-1, in cells treated with ATCC 35896 EVs and purified FtxA, respectively, compared with 148B-17U EVs (p < 0.05) was observed. Control represents untreated THP-1 cells. Data are presented as means ± standard deviation from three independent experiments.

To further validate the immunosuppressive effects of FtxA, a multiplex cytokine array assay was conducted on THP-1 cell culture supernatants after exposure to purified FtxA, and EVs obtained from ATCC 35896 (*ftxA*^+^), and 148B-17U (*ftxA*^−^). Expressions of selected expressed cytokine genes from the array datasets (*i.e.*, IL-8, TNF-α protein and MIP-1α and MIP-1β) were compared with the RNA-Seq data as an internal control to confirm that the approaches were compatible (data not shown). As displayed in **Figure 6** and in **Supplementary Table 5**, the results demonstrated a significant reduction in the secretion of TNF-α, IL-8, IL-10, MIP-1α, MIP-1β, and MCP-1, in cells treated with ATCC 35896 EVs and purified FtxA, compared with 148B-17U EVs (p < 0.05). The suppression of these cytokines, particularly involved in macrophage activation, supports that FtxA could modulate immune responses by inhibiting inflammatory signaling pathways to promote *F. alocis* persistence in periodontitis.

## 4 Discussion

The recently discovered RTX protein of *F. alocis*, FtxA, appears to be associated with both progress and severity of periodontitis (Razooqi et al., 2024b; Razooqi et al., 2024a). Although the underlying mechanism(s) remained unclear, it could be associated with enhanced loads of *F. alocis* (Razooqi et al., 2024b). Here, we investigated mechanistic correlations based on FtxA activity, as present in *F. alocis* cells and extracellular vesicles, and as a recombinant protein, using THP-1 monocytic cells, differentiated to macrophage-like cells, as a model.

### 4.1 FtxA shifts the host response toward immunosuppression

Notably, consistent with neutrophil-dampening activity of *F. alocis* (Ozuna et al., 2022), RNA-Seq revealed an FtxA-dependent downregulation of NF-κB and TNF-α signaling, reduced chemokine and cytokine interactions, and minimal cytokine release. Thus, FtxA here acted to dampen inflammatory signaling, to shift cell signaling status from a pro-inflammatory to an immunosuppressive state. In the periodontal pocket, such immune suppression may explain why FtxA-positive strains are more strongly associated with chronic rather than acute periodontal lesions (Razooqi et al., 2024a). The apparent increase in MALAT*1* expression in THP-1 cells treated with EVs from ATCC 35896 (*ftxA*^+^) suggests that such vesicles can modulate periodontitis pathogenesis, consistent with this LncRNA regulating osteogenic differentiation, proliferation, inflammation, and autophagy of periodontal cells through several pathways (Zhang et al., 2023). In contrast, EVs from 148B-17U (*ftxA*^−^) imposed a strong pro-inflammatory activation with transcriptional upregulation of TNF-α, NF-κB, cytokine–cytokine receptor signaling, with high cytokine release (IL-8, MCP-1, MIP-1α/β, TNF-α), and JAK–STAT activation. JAK-STAT is a key system important for immunity and inflammation and can signal a strong pro-inflammatory response (Hu et al., 2023). Mechanistically, the stronger cytokine production induced by EVs from the *ftxA*^−^ *F. alocis* strain may reflect an increased activation of innate immune sensors (*e.g.*, TLRs), leading to stronger upstream signaling and higher production of proinflammatory cytokines. The concomitant increase in IL-10 upon stimulation with *ftxA*^−^ EVs likely represents a compensatory negative feedback mechanism to limit excessive inflammation (Iyer et al., 2010; Saraiva and O’Garra, 2010). In contrast, decreased IL-10 levels after exposure to FtxA-containing EVs are consistent with a weaker initial immune activation, supporting a model in which FtxA attenuates inflammatory signaling and thus also the downstream IL-10-mediated counterregulatory response. Thus, albeit *F. alocis* could act as a potent immune activator in certain contexts, like co-infection scenarios together with *A. actinomycetemcomitans* (Razooqi et al., 2024b), to promote periodontal inflammation, our present results support an immunosuppressive role of FtxA *per se*.

### 4.2 FtxA as an inhibitor of apoptosis and immune cell recruitment

RNA-seq demonstrated FtxA-dependent downregulated apoptosis- and necroptosis pathways, whereas EVs from the *ftx*A-negative strain 148B-17U strongly upregulated them. Thus, upon extending immune cell delayed apoptosis-based cell lifespans, FtxA may prolong the inflammatory microenvironment in the periodontal pocket to enhance *F. alocis* persistence, immune evasion, and simultaneously sustain inflammation. Similar immune-suppression among RTX toxins includes *Bordetella pertussis* CyaA, paralyzing sentinel phagocytic cells, and *Vibrio cholerae* MARTX, dampening inflammation of intestinal epithelia (Ahmad and Sebo, 2021). Moreover, chicken macrophages exposed to *Gallibacterium anatis* expressing its RTX protein, GtxA, produced high IL-10 (anti‐inflammatory) and little TNF-α/IL-1β, whereas a *gtxA*‐deficient strain induced strong pro‐inflammatory cytokines. Thus, GtxA, similar to FtxA, may host cell responses to an immunosuppressive profile (Tang and Bojesen, 2020). Moreover, suppression of chemokine signaling pathways and leukocyte chemotaxis suggests that FtxA may reduce recruitment of immune cells, further impairing host clearance mechanisms. Blocking immune cell influx and prolonging immune cell survival may create and favor *F. alocis* colonization in oral niche(s).

### 4.3 A potential FtxA-dependent metabolic disruption strategy

The FtxA-associated downregulation of mitochondrial and oxidative phosphorylation pathways in THP-1 cells upon EV stimulation is interesting as mitochondrial dysfunction is increasingly recognized as important in immune modulation, reducing ATP availability, limiting antimicrobial effector functions including ROS generation (Mukherjee et al., 2024). Indeed, *F. alocis* encodes superoxide reductases (i.e., FA51) and additionally ROS defense systems (Mishra et al., 2020), suggesting multiple strategies manipulating redox balance of host cells. Impairing mitochondrial metabolism weakens energy reserves required for sustained immune defense to further promote bacterial survival. In periodontitis, such mitochondrial interference may also affect resident gingival fibroblasts and epithelial cells, compromising barrier function and tissue repair, which could accelerate connective tissue breakdown and alveolar bone resorption (Liu et al., 2022).

## 5 Conclusions

Within the *in vitro* limitation, we show that FtxA can regulate host cell responses, suppressing pro-inflammatory signaling, inhibiting apoptosis and downregulating necroptosis, and disrupting metabolic host metabolism. Such immunosuppressive role might explain why FtxA-expression is associated with progress and severity of periodontitis. To understand molecularly how FtxA affects host cells could therefore enhance novel strategies to mitigate dysbiosis in periodontitis. Therefore, further research is needed to confirm such effects in primary cells, and *in vivo* models.

## Data Availability

All data supporting the findings of the present work are included in this article and its supplementary files. The RNA-sequencing data generated in this study has been deposited in the NCBI Gene Expression Omnibus (GEO) database (http://www.ncbi.nlm.nih.gov/projects/geo) with accession number GSE311337.
