# Supplementary material for: Filifactor alocis FtxA blocks inflammation and apoptosis pathways in monocytic cells
Source: Front Cell Infect Microbiol. 2026 Mar 23;16:1745721. doi: 10.3389/fcimb.2026.1745721 (PMC13050831; doi:10.3389/fcimb.2026.1745721)
Supplement: Supplementary file 1 [file Table1.docx]

**Supplementary Information**

***Filifactor alocis* FtxA blocks inflammation and apoptosis pathways in monocytic cells**

**Zeinab Razooqi^1^, Kai Bao^2^, Abdelbasset Yabrag^3^, Naeem Ullah^3^, Raviprakash T. Sitaram^1^, Mark Lindholm^2^, Mattias Pettersson^1^, Anders Johansson^1^, Georgios N. Belibasakis^2^, Aftab Nadeem^3*^, Jan Oscarsson^1*^**

^1^Department of Odontology, Umeå University, S-90187 Umeå, Sweden

^2^Division of Oral Health and Periodontology, Department of Dental Medicine, Karolinska Institutet, Alfred Nobels Allé 8, 14104 Huddinge, Stockholm, Sweden

^3^Department of Molecular Biology and Umeå Centre for Microbial Research (UCMR), Umeå University, S-90187 Umeå, Sweden

*** Correspondence:**
[jan.oscarsson@umu.se](mailto:jan.oscarsson@umu.se), [aftab.nadeem@umu.se](mailto:aftab.nadeem@umu.se)

**Keywords: *Filifactor alocis*, FtxA, RTX toxin, THP-1 cells, extracellular vesicles, inflammation, apoptosis, periodontitis**

Running title: *Filifactor alocis* FtxA in immune suppression

**
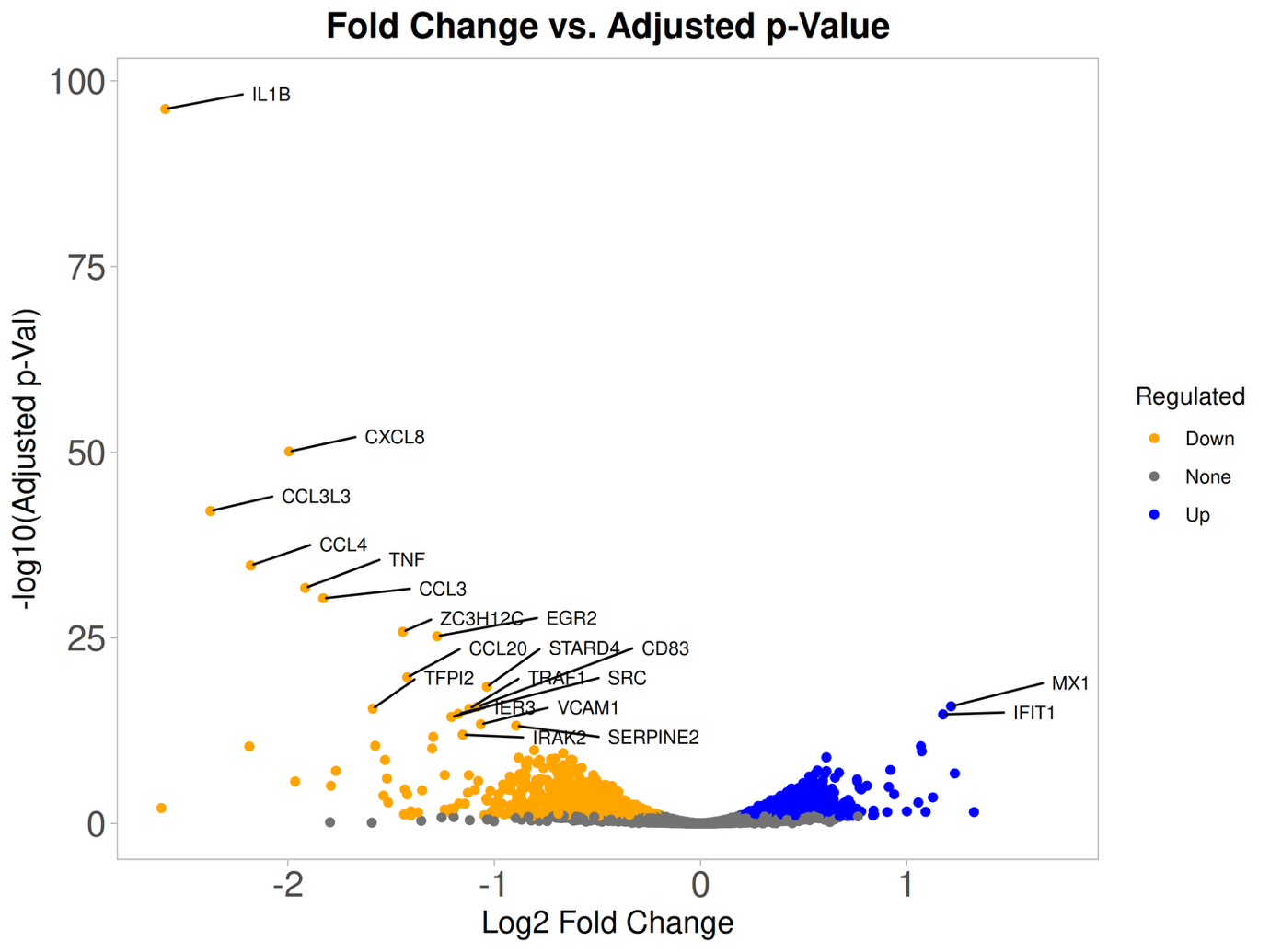
**

**Supplementary Figure 1: Differential expression of genes in THP-1 macrophage-like cells in response to purified FtxA**

THP-1 macrophage-like cells were exposed to purified FtxA (1 μg/ml) for 4 hours. Following RNA extraction, RNA sequencing was performed and the results were analyzed against the control. The top 20 differentially expressed genes (yellow corresponds to downregulated genes, while blue corresponds to upregulated genes) are labelled in the volcano plot.

**Supplementary Table 1.** Identification of prepilin and fimbrial proteins in EVs of *F. alocis* ATCC 35896 (here denoted “Fa1”), and 148B-17U (“Fa12”).

| Protein.Group | unique peptides |
| --- | --- |
| D6GS37;>fig\| 546269.19.peg.268 | 1 |
| D6GS61;>fig\| 546269.19.peg.244 | 29 |
| D6GS62;>fig\| 546269.19.peg.243 | 21 |
| D6GS64;>fig\| 546269.19.peg.241 | 11 |
| D6GRW7;>fig\|546269.15.peg.157;>fig\|546269.10.peg.1391;>fig\|546269.19.peg.341 | 1 |
| D6GS59;>fig\|546269.15.peg.251;>fig\|546269.10.peg.1486;>fig\|546269.19.peg.246 | 3 |
| D6GS63;>fig\|546269.19.peg.242 | 7 |

Proteins that were identified based on shared peptides were grouped into the same protein group and separated by semicolons (“;”). Among these, proteins whose identifiers begin with **“**>**fig|”** were derived from the genomic sequences of *F. alocis* strains included in the custom protein database, where others are UniProt ID. The peptides corresponding to the identified pilin/prepilin proteins are listed below:

| Sample ID | Protein.Group* | Genes.MaxLFQ | Modified.Sequence | Ms1.Area | MS2.Scan |
| --- | --- | --- | --- | --- | --- |
| Fa1_3 | D6GS37 | 126735 | SSADEYSFEITANNNK | 558959 | 40031 |
| Fa1_2 | D6GS37 | 142168 | SSADEYSFEITANNNK | 463007 | 39611 |
| Fa1_3 | D6GS61 | 962351 | AGIFMVDK | 520959 | 42635 |
| Fa1_2 | D6GS61 | 1.03927e+06 | AGIFMVDK | 868321 | 42145 |
| Fa1_3 | D6GS61 | 962351 | AGIFM(UniMod:35)VDK | 543860 | 35636 |
| Fa1_2 | D6GS61 | 1.03927e+06 | AGIFM(UniMod:35)VDK | 684026 | 35216 |
| _Fa1_ | D6GS61 | 28028.7 | ATEITATGSGNEVR | 0 | 20827 |
| Fa1_3 | D6GS61 | 962351 | ATEITATGSGNEVR | 412386 | 20477 |
| Fa1_2 | D6GS61 | 1.03927e+06 | ATEITATGSGNEVR | 757051 | 20407 |
| Fa1_3 | D6GS61 | 962351 | DDYLFREDISIINPNK | 0 | 61771 |
| Fa1_2 | D6GS61 | 1.03927e+06 | DDYLFREDISIINPNK | 0 | 61141 |
| Fa1_3 | D6GS62 | 541212 | AADVELVPFDNNVLVR | 658938 | 65580 |
| Fa1_2 | D6GS62 | 484921 | AADVELVPFDNNVLVR | 1.05216e+06 | 65160 |
| Fa1_3 | D6GS62 | 541212 | AADVELVPFDNNVLVR | 124992 | 65613 |
| Fa1_3 | D6GS63 | 945314 | AGADWAVDLINTNK | 1.58433e+06 | 58562 |
| Fa1_2 | D6GS63 | 1.13757e+06 | AGADWAVDLINTNK | 2.86152e+06 | 57862 |
| Fa1_3 | D6GS63 | 945314 | DDLQLYYYSK | 490028 | 45601 |
| Fa1_2 | D6GS63 | 1.13757e+06 | DDLQLYYYSK | 580262 | 45041 |
| _Fa1_ | D6GS64 | 1.17525e+06 | AAGIVDGQPVGSK | 3.04309e+07 | 23474 |
| Fa1_3 | D6GS64 | 1.33082e+07 | AAGIVDGQPVGSK | 1.05615e+07 | 22214 |
| _Fa12 | D6GS64 | 351964 | AAGIVDGQPVGSK | 0 | 23264 |
| Fa1_2 | D6GS64 | 1.61141e+07 | AAGIVDGQPVGSK | 1.36138e+07 | 22214 |
| _Fa1_ | D6GS64 | 1.17525e+06 | AAGIVDGQPVGSKYVYDGK | 44303.1 | 36990 |
| Fa1_3 | D6GS64 | 1.33082e+07 | AAGIVDGQPVGSKYVYDGK | 666560 | 36500 |
| Fa12_ | D6GS64 | 3.25945e+06 | AAGIVDGQPVGSKYVYDGK | 0 | 36150 |
| Fa1_2 | D6GS64 | 1.61141e+07 | AAGIVDGQPVGSKYVYDGK | 1.65434e+06 | 35940 |
| Fa1_2 | D6GS64 | 1.61141e+07 | DDGMKAIR | 363516 | 15266 |
| Fa1_3 | D6GS62 | 541212 | EESIQEAYASQK | 263336 | 25306 |
| Fa1_2 | D6GS62 | 484921 | EESIQEAYASQK | 121254 | 25096 |
| Fa1_3 | D6GS62 | 541212 | EFLYDLR | 1.19045e+06 | 49709 |
| Fa1_2 | D6GS62 | 484921 | EFLYDLR | 1.15086e+06 | 49149 |
| Fa1_3 | D6GS62 | 541212 | ENNSLNYEMK | 89935.6 | 26277 |
| Fa1_2 | D6GS62 | 484921 | ENNSLNYEMK | 66082.9 | 25997 |
| Fa1_2 | D6GS62 | 484921 | ENNSLNYEM(UniMod:35)K | 232916 | 17738 |
| Fa1_3 | D6GS62 | 541212 | GSPSDPATAIAYR | 1.97668e+06 | 33211 |
| Fa1_2 | D6GS62 | 484921 | GSPSDPATAIAYR | 1.98594e+06 | 32791 |
| Fa1_3 | D6GS62 | 541212 | IFLSYTDNAIK | 1.39022e+06 | 49520 |
| Fa1_2 | D6GS62 | 484921 | IFLSYTDNAIK | 1.57623e+06 | 48890 |
| Fa1_3 | D6GS62 | 541212 | IFQEIGESINNDLWMVNGPK | 214237 | 68016 |
| Fa1_2 | D6GS62 | 484921 | IFQEIGESINNDLWMVNGPK | 0 | 67806 |
| Fa1_3 | D6GS62 | 541212 | IIAFSDLSNGK | 1.25325e+06 | 46082 |
| Fa1_2 | D6GS62 | 484921 | IIAFSDLSNGK | 834363 | 45522 |
| Fa1_3 | D6GS62 | 541212 | IIGSNVYEGK | 1.1555e+06 | 27667 |
| Fa1_2 | D6GS62 | 484921 | IIGSNVYEGK | 1.12812e+06 | 27387 |
| Fa1_3 | D6GS62 | 541212 | LDNSFNFK | 757542 | 37321 |
| Fa1_2 | D6GS62 | 484921 | LDNSFNFK | 1.17448e+06 | 36831 |
| _Fa1_ | D6GS62 | 28270.1 | LSSLNALQVVDR | 38819 | 50712 |
| Fa1_3 | D6GS62 | 541212 | LSSLNALQVVDR | 1.33116e+06 | 50642 |
| Fa1_2 | D6GS62 | 484921 | LSSLNALQVVDR | 1.41108e+06 | 50012 |
| Fa1_3 | D6GS62 | 541212 | LTPDWNYIGLK | 1.37162e+06 | 59112 |
| Fa1_2 | D6GS62 | 484921 | LTPDWNYIGLK | 1.327e+06 | 58482 |
| Fa1_3 | D6GS62 | 541212 | LYNTPDNYLK | 478769 | 37267 |
| Fa1_2 | D6GS62 | 484921 | LYNTPDNYLK | 731040 | 36777 |
| Fa1_2 | D6GS62 | 484921 | NTNPIYR | 503986 | 18554 |
| Fa1_3 | D6GS62 | 541212 | NYDMDAFQK | 76931.5 | 34390 |
| Fa1_2 | D6GS62 | 484921 | NYDMDAFQK | 114200 | 33970 |
| Fa1_3 | D6GS62 | 541212 | NYDM(UniMod:35)DAFQK | 411044 | 22701 |
| Fa1_2 | D6GS62 | 484921 | NYDM(UniMod:35)DAFQK | 356476 | 22561 |
| Fa1_3 | D6GS62 | 541212 | PTFYSASK | 589752 | 21846 |
| Fa1_2 | D6GS62 | 484921 | PTFYSASK | 519121 | 21846 |
| Fa1_3 | D6GS62 | 541212 | SVSTSGLNAIADEFSIK | 715306 | 66278 |
| Fa1_2 | D6GS62 | 484921 | SVSTSGLNAIADEFSIK | 621257 | 65998 |
| Fa1_3 | D6GS62 | 541212 | TALVAIDYK | 749450 | 41242 |
| Fa1_2 | D6GS62 | 484921 | TALVAIDYK | 521296 | 40822 |
| Fa1_2 | D6GS62 | 484921 | TDNVYLK | 405389 | 21143 |
| Fa1_3 | D6GS62 | 541212 | VYIIAFSR | 229268 | 52160 |
| Fa1_2 | D6GS62 | 484921 | VYIIAFSR | 243035 | 51530 |
| Fa1_3 | D6GS62 | 541212 | YAFYSIDEK | 850829 | 42790 |
| Fa12_ | D6GS62 | 32195.7 | YAFYSIDEK | 0 | 42160 |
| Fa1_2 | D6GS62 | 484921 | YAFYSIDEK | 766481 | 42230 |
| _Fa1_ | D6GS64 | 1.17525e+06 | KLTVTTTSPNEYSSPLPTLEYDFTN | 2.10051e+06 | 66847 |
| Fa1_3 | D6GS64 | 1.33082e+07 | KLTVTTTSPNEYSSPLPTLEYDFTN | 1.2048e+08 | 66427 |
| Fa12_ | D6GS64 | 20997.1 | KLTVTTTSPNEYSSPLPTLEYDFTN | 200058 | 66427 |
| _Fa12 | D6GS64 | 351964 | KLTVTTTSPNEYSSPLPTLEYDFTN | 5.345e+06 | 66427 |
| Fa1_2 | D6GS64 | 1.61141e+07 | KLTVTTTSPNEYSSPLPTLEYDFTN | 1.36899e+08 | 66147 |
| Fa1_2 | D6GS64 | 1.61141e+07 | KQGVLPSK | 580248 | 13163 |
| Fa1_2 | D6GS64 | 1.61141e+07 | KSANTSVLQTNYK | 169375 | 21920 |
| Fa1_3 | D6GS64 | 1.33082e+07 | LTVTTTSPNEYSSPLPTLEYDFTN | 6.17918e+06 | 68242 |
| Fa1_2 | D6GS64 | 1.61141e+07 | LTVTTTSPNEYSSPLPTLEYDFTN | 7.27681e+06 | 68102 |
| Fa1_3 | D6GS64 | 1.33082e+07 | QGVLPSKDDGMK | 0 | 22149 |
| Fa1_2 | D6GS64 | 1.61141e+07 | QGVLPSKDDGMK | 117704 | 22009 |
| Fa1_3 | D6GS64 | 1.33082e+07 | QGVLPSKDDGMK | 289395 | 22123 |
| Fa1_2 | D6GS64 | 1.61141e+07 | QGVLPSKDDGMK | 1.72756e+06 | 21983 |
| Fa1_2 | D6GS64 | 1.61141e+07 | QGVLPSKDDGM(UniMod:35)K | 182183 | 16200 |
| Fa1_2 | D6GS64 | 1.61141e+07 | QGVLPSKDDGM(UniMod:35)K | 3.47316e+06 | 16173 |
| Fa1_3 | D6GS64 | 1.33082e+07 | SANTSVLQTNYK | 770144 | 25862 |
| Fa1_2 | D6GS64 | 1.61141e+07 | SANTSVLQTNYK | 2.33104e+06 | 25652 |
| Fa1_3 | D6GS64 | 1.33082e+07 | TC(UniMod:4)ISVINLEMAK | 82152.3 | 57856 |
| _Fa12 | D6GS64 | 351964 | TC(UniMod:4)ISVINLEMAK | 96129.3 | 57646 |
| Fa1_2 | D6GS64 | 1.61141e+07 | TC(UniMod:4)ISVINLEMAK | 0 | 57226 |
| _Fa1_ | D6GS64 | 1.17525e+06 | TC(UniMod:4)ISVINLEM(UniMod:35)AK | 3.16609e+06 | 48197 |
| Fa1_3 | D6GS64 | 1.33082e+07 | TC(UniMod:4)ISVINLEM(UniMod:35)AK | 3.44722e+07 | 48057 |
| Fa1_2 | D6GS64 | 1.61141e+07 | TC(UniMod:4)ISVINLEM(UniMod:35)AK | 3.05458e+07 | 47497 |
| Fa1_2 | D6GS64 | 1.61141e+07 | YVYDGKK | 133007 | 11834 |
| Fa12_ | D6GRW7 | 47970.9 | SVSIDEVLTTDGK | 542262 | 47635 |
| Fa12_ | D6GRW7 | 38880.2 | SVSIDEVLTTDGK | 869917 | 47845 |
| _Fa12 | D6GRW7 | 28888.2 | SVSIDEVLTTDGK | 1.22485e+06 | 47705 |
| Fa12_ | D6GS59 | 30865.4 | DFNFFAPYLK | 123020 | 69258 |
| Fa12_ | D6GS59 | 27057.1 | DFNFFAPYLK | 639341 | 69258 |
| Fa1_2 | D6GS59 | 41236.8 | DFNFFAPYLK | 250284 | 69118 |
| Fa1_3 | D6GS59 | 38030.2 | DLYLNDIAIQNR | 0 | 53100 |
| Fa12_ | D6GS59 | 30865.4 | DLYLNDIAIQNR | 0 | 52960 |
| Fa12_ | D6GS59 | 27057.1 | DLYLNDIAIQNR | 0 | 53100 |
| _Fa12 | D6GS59 | 32642 | DLYLNDIAIQNR | 0 | 52890 |
| Fa1_2 | D6GS59 | 41236.8 | DLYLNDIAIQNR | 0 | 52470 |
| Fa1_3 | D6GS59 | 38030.2 | HAIAQFER | 40636 | 20660 |
| Fa1_2 | D6GS59 | 41236.8 | HAIAQFER | 50742.1 | 20450 |
| _Fa1_ | D6GS63 | 15152.5 | EGLDITGGSGR | 0 | 28296 |
| Fa1_3 | D6GS63 | 945314 | EGLDITGGSGR | 562307 | 28226 |
| Fa1_2 | D6GS63 | 1.13757e+06 | EGLDITGGSGR | 2.11139e+06 | 28016 |
| Fa1_3 | D6GS63 | 945314 | NNELDPSGTIER | 737888 | 29294 |
| Fa1_2 | D6GS63 | 1.13757e+06 | NNELDPSGTIER | 932210 | 28944 |
| Fa1_3 | D6GS63 | 945314 | SLLEEIK | 1.05658e+06 | 41791 |
| Fa1_2 | D6GS63 | 1.13757e+06 | SLLEEIK | 1.2687e+06 | 41231 |
| Fa1_3 | D6GS63 | 945314 | VGHFEVEVDK | 0 | 28932 |
| Fa1_2 | D6GS63 | 1.13757e+06 | VGHFEVEVDK | 81723.7 | 28512 |
| Fa1_3 | D6GS63 | 945314 | VISTGSYAVGTK | 845197 | 26763 |
| Fa1_2 | D6GS63 | 1.13757e+06 | VISTGSYAVGTK | 837455 | 26483 |

*The UniProt ID in each protein group Ids were used as presentative for each protein groups.

**Supplementary Table 2.** Identification of FtxA in EVs of ATCC 35896 (3 peptides identified; sample ID here denoted ”Fa1”). The peptides with a +2 charge identified from all samples were derived from E8RK95 and >fig|546269.19.peg.785. E8RK95 is the UniProt ID corresponding to FtxA, whereas >fig|546269.19.peg.785 is derived from the genomic sequences of *F. alocis* strain. Both represent FtxA and share identical sequences.

| Sample ID | Genes.MaxLFQ | Modified.Sequence | Ms1.Area | MS2.Scan |
| --- | --- | --- | --- | --- |
| Fa1_3 | 48919.5 | DIFNFISR | 44853.1 | 64763 |
| Fa1_2 | 69162 | DIFNFISR | 224235 | 64203 |
| Fa1_3 | 48919.5 | IAYDLILTSK | 38350.4 | 52731 |
| Fa1_2 | 69162 | IAYDLILTSK | 0 | 52101 |
| Fa1_3 | 48919.5 | IFAITNNDLGEDVEK | 72182.3 | 51014 |
| Fa1_2 | 69162 | IFAITNNDLGEDVEK | 89496 | 50384 |

**Supplementary Table 3.** Biological processes as identified based on RNA-Seq data in THP-1 cells treated with EVs from *F. alocis* 148B-17U (*ftxA*^-^), ATCC 35896 (*ftxA*^+^), and FtxA holotoxin, respectively, and comparing all three stimuli in the last section.

| F.alocis 148B-17U | Enrichment Score | NES | pvalue | core_enrichment genes |
| --- | --- | --- | --- | --- |
| activation of cysteine-type endopeptidase activity involved in apoptotic process | 0.57 | 1.35 | 0.12 | STAT1; NKX3-1; PMAIP1 |
| apoptotic mitochondrial changes | 0.5 | 1.17 | 0.3 | PIM2; PIM2-2 |
| apoptotic process | 0.4 | 1.41 | 0.03 | IL1A; NR4A1; XAF1; TNFRSF4; BIRC3; TNFSF15; FAS; IRF1; CSRNP1; KLLN-2; TNFRSF9; FOXO3B; BCL3; G0S2; TP53BP2; XIAP; TICAM2; PLAGL2; SRGN; ZMAT1; NOTCH1; EP300; BMF; PPP1R15A;NewGene_3651; C5AR1; PRKCD; BCL2L13; HIP1; PERP; DIDO1; BID; BIRC6; TOPORS; ZFP36L2; TNFRSF10D; POU4F2; PAWR; BCLAF1 |
| apoptotic signaling pathway | 0.51 | 1.51 | 0.03 | IL1A; TNFRSF4; TNFRSF9; FOXO3B; BCL3; G0S2; TP53BP2; TICAM2; SRGN; ZMAT1; EP300; PPP1R15A; MLLT11; PERP; DIDO1; TOPORS; TNFRSF10D; POU4F2 |
| cell death | 0.44 | 1.46 | 0.03 | IL1A; TNFRSF4; BIRC3; CSRNP1; KLLN-2; TNFRSF9; FOXO3B; SUSD6; BCL3; G0S2; TP53BP2; XIAP; TICAM2; PLAGL2; SRGN; ZMAT1; NOTCH1; EP300; PPP1R15A |
| extrinsic apoptotic signaling pathway | 0.67 | 1.53 | 0.05 | IL1A; FOXO3B; G0S2 |
| extrinsic apoptotic signaling pathway in absence of ligand | 0.66 | 1.53 | 0.04 | IL1A; FOXO3B; KITLG; ITGAV; BCL2 |
| intrinsic apoptotic signaling pathway | 0.29 | 0.81 | 0.72 | BCL3; TP53BP2; ZMAT1; EP300; PPP1R15A; PERP; TOPORS; TNFRSF10D; POU4F2 |
| intrinsic apoptotic signaling pathway by p53 class mediator | 0.43 | 1.01 | 0.47 | BCL3; TP53BP2; ZMAT1; EP300; PERP; TOPORS; POU4F2 |
| intrinsic apoptotic signaling pathway in response to DNA damage | 0.25 | 0.65 | 0.91 | BCL3; BCL2; EP300; TOPORS; BCL2L1; ATM; SIRT1 |
| intrinsic apoptotic signaling pathway in response to DNA damage by p53 class mediator | 0.51 | 1.17 | 0.28 | BCL3; TP63; DDIT4; EP300; CDKN1A |
| programmed cell death | 0.44 | 1.43 | 0.04 | IL1A; TNFRSF4; BIRC3; CSRNP1; KLLN-2; TNFRSF9; FOXO3B; BCL3; G0S2; TP53BP2; XIAP; TICAM2; PLAGL2; SRGN; ZMAT1; NOTCH1; EP300; PPP1R15A |

| F.alocis ATCC35896 | enrichmentScore | NES | pvalue | core_enrichment genes |
| --- | --- | --- | --- | --- |
| activation of cysteine-type endopeptidase activity involved in apoptotic process | -0.53 | -1.48 | 0.06 | TNFRSF10D; DAP; NGFR; IFT57; MTCH1; BEX3; NKX3-1; DIABLO; BID; CRADD |
| apoptotic mitochondrial changes | -0.4 | -1.09 | 0.37 | AKT1; ATG3; AIFM2; STPG1; PIM2; PPP2CB; CLU; FIS1; PIM2-2; SLC25A4 |
| apoptotic process | -0.3 | -1.22 | 0.15 | TNFRSF6B; IFT57; SHARPIN; NUDT2; PERP; DDIT3; NAIF1; CLU; CIDEB-2; IRF1; TNFRSF9; BEX3; C5AR1; FIS1; POLR2G; BIK; DIABLO; MAGEH1; CASP9; CST3; PHLDA3; BID; PHLDA2-2; SIVA1; NME3; G0S2; MTFP1;NewGene_10785; KLLN; BAG6-5; BCL3; ZNF442; PHLDA2; TNFRSF4; BAG6-3 |
| apoptotic signaling pathway | -0.34 | -1.17 | 0.23 | PHLDA3; G0S2; BAG6-5; BCL3; TNFRSF4; BAG6-3 |
| cell death | -0.34 | -1.3 | 0.11 | PHLDA3; PHLDA2-2; NME3; G0S2;NewGene_10785; KLLN; BAG6-5; BCL3; ZNF442; PHLDA2; TNFRSF4; BAG6-3 |
| extrinsic apoptotic signaling pathway | -0.39 | -0.97 | 0.5 | BEX3; G0S2; WWOX |
| extrinsic apoptotic signaling pathway in absence of ligand | 0.31 | 0.82 | 0.69 | FOXO3B; FOXO3; ITGAV; KITLG; BCL2 |
| intrinsic apoptotic signaling pathway | -0.48 | -1.56 | 0.03 | PHLDA3; BBC3; HRAS; BAG6-5; BCL3; BAG6-3 |
| intrinsic apoptotic signaling pathway by p53 class mediator | -0.61 | -1.69 | 0.02 | PHLDA3; WWOX; BAG6-5; BCL3; BAG6-3 |
| intrinsic apoptotic signaling pathway in response to DNA damage | -0.36 | -1.08 | 0.34 | BAD; PHLDA3; BAG6-5; BCL3; BAG6-3 |
| intrinsic apoptotic signaling pathway in response to DNA damage by p53 class mediator | -0.73 | -1.83 | 0 | TP53; CDKN1A; DDIT4; PHLDA3; BAG6-5; BCL3; BAG6-3 |
| programmed cell death | -0.35 | -1.32 | 0.1 | PHLDA3; PHLDA2-2; NME3; G0S2;NewGene_10785; KLLN; BAG6-5; BCL3; ZNF442; PHLDA2; TNFRSF4; BAG6-3 |

| FtxA | enrichmentScore | NES | pvalue | core_enrichment genes |
| --- | --- | --- | --- | --- |
| activation of cysteine-type endopeptidase activity involved in apoptotic process | -0.33 | -0.88 | 0.61 | NGFR; TNFRSF10D; NKX3-1; BCL2L11 |
| apoptotic mitochondrial changes | -0.29 | -0.78 | 0.77 | MAP3K1; PPP2CB; BCL2L11; PIM2-2 |
| apoptotic process | -0.33 | -1.37 | 0.06 | SH3RF1; PDCD6IP; SAV1; NGFR; XIAP; DAPK1; TOPORS; PPP2R1B; TNFRSF6B;NewGene_4132; PERP; TGFBR2; SGPP1; BCL3; BIRC6; BCLAF1P2; ATN1; SGPP1-2; OXR1; JMY; TNFRSF10D; FOXO3B; TNFSF15; PLAGL2; C5AR1;NewGene_3651; BCL2L11; PAK5; HIP1; TNFRSF9; TNFRSF4; FNIP2; ZFP36L1; NCKAP1; BIRC3; EP300 |
| apoptotic signaling pathway | -0.39 | -1.34 | 0.11 | FOXO3; PPP1R15A; TP53BP2; POU4F2; CASP8AP2-2; NGFR; TOPORS; TNFRSF6B; PERP; SGPP1; BCL3; SGPP1-2; JMY; TNFRSF10D; FOXO3B; BCL2L11; TNFRSF9; TNFRSF4; FNIP2; EP300 |
| cell death | -0.34 | -1.31 | 0.09 | NGFR; XIAP; TOPORS; PPP2R1B; TNFRSF6B;NewGene_4132; PERP; SGPP1; BCL3; ATN1; SGPP1-2; OXR1; JMY; TNFRSF10D; FOXO3B; PLAGL2; C5AR1;NewGene_3651; BCL2L11; TNFRSF9; TNFRSF4; DSG2; FNIP2; NCKAP1; BIRC3; EP300 |
| extrinsic apoptotic signaling pathway | -0.69 | -1.69 | 0.02 | SGPP1; WWOX; SGPP1-2; TNFRSF10D; FOXO3B; BCL2L11 |
| extrinsic apoptotic signaling pathway in absence of ligand | -0.51 | -1.3 | 0.16 | BCL2; FOXO3; KITLG; CASP2; FOXO3B; ITGAV; BCL2L11 |
| intrinsic apoptotic signaling pathway | -0.37 | -1.16 | 0.24 | TOPORS; PERP; SGPP1; BCL3; SGPP1-2; JMY; TNFRSF10D; BCL2L11; FNIP2; EP300 |
| intrinsic apoptotic signaling pathway by p53 class mediator | -0.43 | -1.17 | 0.27 | TP53BP2; POU4F2; TOPORS; PERP; WWOX; BCL3; JMY; EP300 |
| intrinsic apoptotic signaling pathway in response to DNA damage | -0.38 | -1.1 | 0.3 | PIK3R1; SIRT1; TOPORS; BCL3; BCL2L11; ATM; FNIP2; EP300 |
| intrinsic apoptotic signaling pathway in response to DNA damage by p53 class mediator | -0.5 | -1.26 | 0.19 | CDKN1A; TOPORS; BCL3; EP300; TP63 |
| programmed cell death | -0.37 | -1.44 | 0.05 | PDCD6IP; NGFR; XIAP; TOPORS; PPP2R1B; TNFRSF6B;NewGene_4132; PERP; SGPP1; BCL3; ATN1; SGPP1-2; OXR1; JMY; TNFRSF10D; FOXO3B; PLAGL2; C5AR1;NewGene_3651; BCL2L11; TNFRSF9; TNFRSF4; DSG2; FNIP2; NCKAP1; BIRC3; EP300 |

|  | *F. alocis* 148B-17U | *F. alocis* ATCC35896 | FtxA |
| --- | --- | --- | --- |
| extrinsic apoptotic signaling pathway in absence of ligand | 1.53 | 0.82 | -1.3 |
| activation of cysteine-type endopeptidase activity involved in apoptotic process | 1.35 | -1.48 | -0.88 |
| apoptotic mitochondrial changes | 1.17 | -1.09 | -0.78 |
| apoptotic process | 1.41 | -1.22 | -1.37 |
| apoptotic signaling pathway | 1.51 | -1.17 | -1.34 |
| cell death | 1.46 | -1.3 | -1.31 |
| extrinsic apoptotic signaling pathway | 1.53 | -0.97 | -1.69 |
| intrinsic apoptotic signaling pathway | 0.81 | -1.56 | -1.16 |
| intrinsic apoptotic signaling pathway by p53 class mediator | 1.01 | -1.69 | -1.17 |
| intrinsic apoptotic signaling pathway in response to DNA damage | 0.65 | -1.08 | -1.1 |
| intrinsic apoptotic signaling pathway in response to DNA damage by p53 class mediator | 1.17 | -1.83 | -1.26 |
| programmed cell death | 1.43 | -1.32 | -1.44 |

**Supplementary Table 4.** KEGG pathways as identified based on RNA-Seq data in THP-1 cells treated with EVs from *F. alocis* 148B-17U (*ftxA*^-^), ATCC 35896 (*ftxA*^+^), and FtxA holotoxin, respectively, and comparing all three stimuli in the last section.

***F. alocis* 148B-17U**

| Description | enrichmentScore | NES | pvalue | core_enrichment genes |
| --- | --- | --- | --- | --- |
| Necroptosis | 0.54 | 1.91 | 0 | IL1A; TNFSF10; STAT4; IL1B; IL33; STAT1; STAT2; EIF2AK2; BIRC3; CFLAR; IRF9-2; STAT5A; TLR3; TNFAIP3; IRF9; FAS; NLRP3-2; CYLD; TNFRSF10B; JAK2; MLKL; NLRP3; TTC9; XIAP; SQSTM1-2; TICAM2; RNF31-2; CHMP5; BIRC2; CAMK2A; TYK2; RIPK1; TRADD; BCL2; TLR4; TICAM1; RNF31; STAT6; JAK1; STAT3; FTH1; CYBB; PLA2G4C; CASP1 |
| Apoptosis | 0.4 | 1.41 | 0.03 | TNFSF10; TRAF1; BIRC3; CFLAR; BCL2A1; NFKB1; PTPN13; FAS; TNFRSF10B; NFKBIA; CASP10; CSF2RB; GADD45B; PIK3CD; EIF5A-2; XIAP; CTSO-2; JUN; BIRC2; MAP3K5; PARP4; RIPK1; TRADD; BCL2; MCL1; ERN1; DAB2IP; CASP7; ITPR2; DAXX-4; BID; AKT3-2; TNFRSF10A; IL3RA-2; PDPK1; ITPR3; CAPN2; CTSS; BCL2L1; PIK3R3; NRAS |

***F. alocis* ATCC 35896**

| Description | enrichmentScore | NES | pvalue | core_enrichment genes |
| --- | --- | --- | --- | --- |
| Necroptosis | -0.32 | -1.3 | 0.1 | SQSTM1-2; SLC25A5; NLRP3; SLC25A6; VDAC2; GLUD2; TTC9; SPATA2; SLC25A6-2; CHMP4B; SLC25A4; PYCARD; TRADD; USP21; TICAM1; CAMK2A; SPATA2L; PPIA; RIPK3-2; H2AZ1; RNF31-2; FTL; BAX; TMEM42-2; H2AC18; BID; IFNGR2; H2AC25-2; IRF9-2; PLA2G4C; CHMP4A-2; H2AC25; TMEM42; FTH1; H2AJ; SQSTM1; IL1B; ZNF771 |
| Apoptosis | -0.32 | -1.34 | 0.07 | DAXX-3; CTSB; BAK1; ACTG1-2; GADD45B; CTSZ; ACTG1; NTRK1; DDIT3; MAPK3; NFKB1; TUBA4A; TUBA1B; TUBA1C; CTSD; RELA; CTSW; AKT3; HTRA2; ACTB; TP53; GADD45A; TRADD; ENDOG; CTSH; DIABLO; DAB2IP; CTSK; PARP2; TRAF1; BAX; CASP9; BAD; BID; BBC3; GADD45G; BCL2A1; HRAS; NFKBIA; IL3RA; EIF5A; CTSO; PRF1 |

**FtxA holotoxin**

| Description | enrichmentScore | NES | pvalue | core_enrichment genes |
| --- | --- | --- | --- | --- |
| Necroptosis | -0.25 | -1.01 | 0.43 | SQSTM1-2; MAPK8; TNFRSF10B; H2AC25; H2AC25-2; TRPM7; JAK2; STAT5A; NLRP3-2; XIAP; ALOX15; IFNGR2;NewGene_4132; H2AC6; SNTB1; CHMP4A-2; JAK1; CFLAR; TLR4; SNTB2; TNFAIP3; BIRC3;NewGene_4204; GLUD2; TOX; CAMK2A; IL1B |
| Apoptosis | -0.2 | -0.83 | 0.8 | CTSO-2; MAPK8; MAP2K1; TNFRSF10B; CHUK; KRAS; CASP2; ITPR2; XIAP; PARP4; NFKB1; ITPR1; CTSK; ERN1; EIF2AK3; CFLAR; ARPP19; DAB2IP; BCL2L11; MAP3K5; PRF1; PDPK1; AKT3; BCL2A1; ATM; BIRC3; TRAF1 |


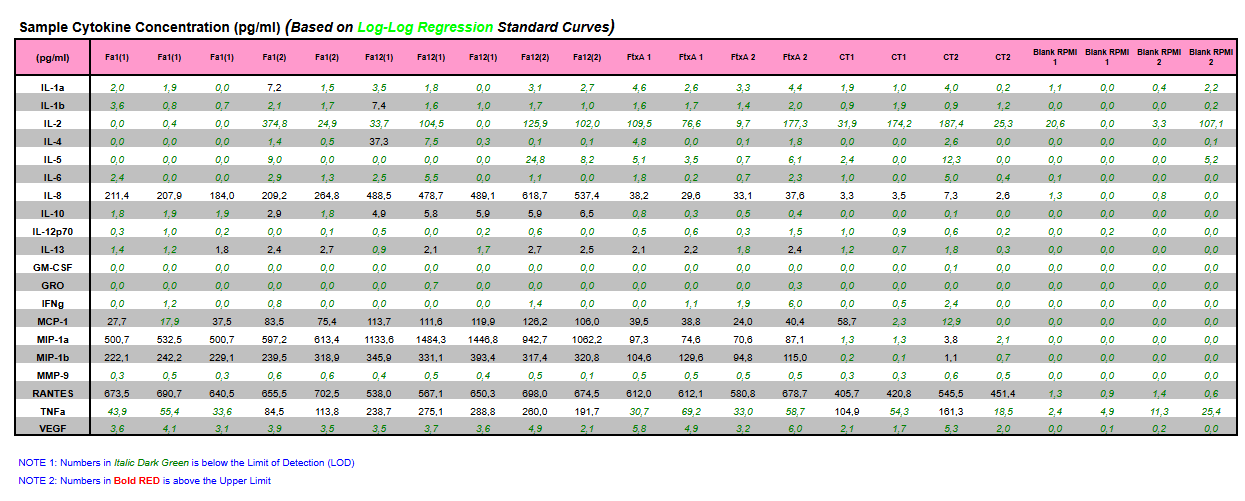
**Supplementary Table 5.** Data from the multiplex cytokine assay. Shown is data (triplicates) on THP-1 cells treated with EVs from *F. alocis* ATCC 35896 (*ftxA*^+^; “FA1”), 148B-17U (*ftxA*^-^; “FA12”), and FtxA holotoxin (“FtxA”), respectively.
